# Validation of prediction models based on lasso regression with multiply imputed data

**DOI:** 10.1186/1471-2288-14-116

**Published:** 2014-10-16

**Authors:** Jammbe Z Musoro, Aeilko H Zwinderman, Milo A Puhan, Gerben ter Riet, Ronald B Geskus

**Affiliations:** >Department of Clinical Epidemiology, Biostatistics and Bioinformatics, Academic Medical Center, University of Amsterdam, Meibergdreef 9, 1105 Amsterdam, the Netherlands; Department of General Practice, Academic Medical Center, University of Amsterdam, Meibergdreef 9, 1105 Amsterdam, the Netherlands; Institute for Social and Preventive Medicine, University of Zurich, Hirschengraben 84, CH 8001 Zurich, Switzerland

**Keywords:** Clinical prediction models, Model validation, Multiple imputation, Quality of life, Shrinkage

## Abstract

**Background:**

In prognostic studies, the lasso technique is attractive since it improves the quality of predictions by shrinking regression coefficients, compared to predictions based on a model fitted via unpenalized maximum likelihood. Since some coefficients are set to zero, parsimony is achieved as well. It is unclear whether the performance of a model fitted using the lasso still shows some optimism. Bootstrap methods have been advocated to quantify optimism and generalize model performance to new subjects. It is unclear how resampling should be performed in the presence of multiply imputed data.

**Method:**

The data were based on a cohort of Chronic Obstructive Pulmonary Disease patients. We constructed models to predict Chronic Respiratory Questionnaire dyspnea 6 months ahead. Optimism of the lasso model was investigated by comparing 4 approaches of handling multiply imputed data in the bootstrap procedure, using the study data and simulated data sets. In the first 3 approaches, data sets that had been completed via multiple imputation (MI) were resampled, while the fourth approach resampled the incomplete data set and then performed MI.

**Results:**

The discriminative model performance of the lasso was optimistic. There was suboptimal calibration due to over-shrinkage. The estimate of optimism was sensitive to the choice of handling imputed data in the bootstrap resampling procedure. Resampling the completed data sets underestimates optimism, especially if, within a bootstrap step, selected individuals differ over the imputed data sets. Incorporating the MI procedure in the validation yields estimates of optimism that are closer to the true value, albeit slightly too larger.

**Conclusion:**

Performance of prognostic models constructed using the lasso technique can be optimistic as well. Results of the internal validation are sensitive to how bootstrap resampling is performed.

**Electronic supplementary material:**

The online version of this article (doi:10.1186/1471-2288-14-116) contains supplementary material, which is available to authorized users.

## Background

The least absolute shrinkage and selection operator (lasso) [1] is a popular technique for model selection and estimation in linear regression models. For a traditional generalized linear regression model, the coefficients *β*_0_ and *β* = (*β*_1_,*β*_2_,…,*β*_*P*_) are estimated by
1

where *Y* and *X* are the outcome and predictors respectively. *λ* is a non-negative tuning parameter that controls the amount of shrinkage, with increased shrinkage for higher *λ* values. The optimal *λ* based on some criterion, for instance mean-squared error (MSE), can be estimated in a generalized cross-validation procedure [2] or via bootstrapping [3]. In prognostic studies, the lasso is particularly appealing for its ability to shrink regression coefficients and automatically perform variable selection by setting some coefficients to zero. This improves predictive performance and introduces parsimony. Models with fewer predictor variables are usually easier to implement in practice and therefore we are sometimes willing to sacrifice some predictive performance. For instance most clinicians and primary care physicians in particular will be unwilling to use large modes that require the collection of too much information. Whether or not the amount of parsimony is satisfactory depends on the model performance as well as its interpretability and practical usefulness. It could be argued that because of the inherent shrinkage, the lasso is free of optimism. However it is unclear if such is the case. Thus, our first aim was to check optimism in the predictive value of a lasso model through some form of model validation.

Validation of prognostic models is paramount in ensuring generalizability to new data [4]. A traditional approach is to split data and perform model development (*training*) on a sample, and model validation (*test*) on the remainder. Any discrepancy between the *training* (apparent performance) and the *test* performance is considered as evidence of optimism. However, there is a substantial loss of estimation precision from models developed on a subset of the data [5, 6]. Alternatively, bootstrapping procedures that make full use of the data and give nearly unbiased estimates of future model performance have been advocated [5, 7–9]. These procedures internally validate the original model fitting process and provide an estimate of the expected value of the optimism. In the same procedure, a shrinkage factor that adapts parameters to improve predictive performance [10, 11] can be estimated.

Missing data are common in prognostic studies. Multiple imputation (MI) has been recommended to account for the uncertainty caused by the missing data. Assuming that the incomplete data is missing at random (MAR) and correct imputation models are used, usually 5 to 10 imputations are enough to yield correct statistical inference [12, 13]. Current guidance recommends that one imputation should be done per percent of incomplete observations [14]. Nevertheless, handling the multiply imputed data sets in the model development and validation process poses an extra challenge, and some strategies to go about this have been discussed in the literature [12, 15, 16].

Vergouw et al. [16] and Heymans et al. [12] combined MI with backward elimination (BE) and bootstrapping to obtain a parsimonious prediction model. However, the authors did not describe how the multiply imputed data sets were handled in the validation procedure. Our second goal was to investigate how internal validation should be applied in the presence of multiply imputed data sets. Two scenarios were considered; (*i*) the data sets that had been completed via MI were resampled, and a pertinent question was: for every bootstrap draw, how should subjects be sampled across the imputed data sets? Should they be the same across the imputed data sets or should separate bootstrap samples be drawn from every imputed data set? (*ii*) the incomplete data was resampled and then MI was performed, thus incorporating the MI procedure in the validation. Although the latter approach is expected to perform better, it is more time consuming and we also wanted to investigate methods that prevent extra imputations.

In this paper, we constructed models to predict Chronic Respiratory Questionnaire (CRQ) dyspnea 6 months ahead using data from a cohort study on Chronic Obstructive Pulmonary Disease (COPD) patients. We investigated optimism of the lasso model via bootstrap resampling, and evaluated four approaches of handling multiply imputed data in the resampling procedure on both the study data and simulated data sets.

The rest of this paper is organized as follows. First we describe the study data, and then enumerate the various steps to construct and validate our models in the presence of multiply imputed data. Second, we show results from comparing four approaches in handling multiply imputed data when quantifying optimism via bootstrapping. Third, a simulation study that further investigated the differences between the four approaches is presented. We end with a discussion.

## Methods

### The study data

The data were based on an international prospective cohort study on COPD patients. A total of 409 primary care COPD patients from Switzerland and the Netherlands were recruited. At entry all patients had GOLD stage II-IV (66%, 25% and 9% respectively), were aged ≥ 40, had GOLD stage A-D (41%, 21%, 15% and 23% respectively), and had been free of exacerbations for at least four weeks. The mean age was 67 years. Patients were contacted by telephone every 6 months within a 5 years follow-up period. The study has been approved by all local medical ethics committees (Academic Medical Center, University of Amsterdam, The Netherlands; Kanton of Zurich, Switzerland and Kanton of St Gallen, Switzerland) and all patients provided written informed consent. For an elaborated description of the study design and the baseline characteristics of the patients see [17, 18].

#### Outcome measures

The outcome was quality of life (QoL) dyspnea as measured by the CRQ at 12 months after entry. Questionnaires were self-administered [19, 20] and consisted of 20 questions. The summary score was on a 7-point scale, where 1 indicates the worst and 7 the best possible score. We applied a penalized linear regression since the outcome did not have a very skewed distribution. Alternatively, an ordinal regression can be considered as well since the outcome measure is ordinal, but with many levels.

#### Candidate predictors

All predictors were selected on the basis of their practicality and suspected prognostic value in primary care. Some predictors were updated at subsequent visits. Forty five predictors were initially considered. This included previous CRQ dyspnea, fatigue, emotional function and mastery along with their change scores (change between baseline and 6 months data). A detailed description of all candidate predictors and the data at baseline was published previously [17, 18]. There was more missing data at 6 months (for those covariates that changed over time) compared to baseline. Thus, in order to investigate the effect of missing data, we used covariates collected at 6 months to predict the outcome at 12 months. Only patients who were still alive after 12 months (*n* = 387) were included.

#### Missing data

Among the predictors and the outcome variable, data were missing in the range of 0 to 19%. All missing data were multiply imputed via the Multivariate Imputation by Chained Equations (MICE) procedure [21]. The imputation model was adapted to the type of outcome. Incomplete dichotomous variables were imputed using a logistic regression model, while predictive mean matching was used to impute incomplete continuous variables. A linear multilevel model was applied for incomplete continuous variables that changed over time (though in the analysis only information collected at 6 months was used to predict CRQ dyspnea 6 months ahead). All available data, including the outcome variable, were used in the imputation models [22]. We generated 10 imputed data sets. Assuming MAR, using imputed outcome values in the analysis can add needless noise to estimates. This is true for estimating parameters that govern the conditional distribution of the outcome given the covariates [23]. Hence, except for the null model, all imputed outcome values (14%) were excluded from the rest of the analysis.

### Variable selection, model fitting, performance and validation

#### Variable selection and model fitting via the lasso

The optimal penalty tuning parameter of the lasso *λ* was chosen, separately for each imputed data set, from a grid of 40 penalty values. For each penalty value a bootstrap corrected MSE was computed as follows. A model was constructed on a bootstrap sample (drawn randomly with replacement from the original data set, and of the same size as the original data set), followed by a comparison of the observed and predicted values in the original imputed data set using the constructed model. This was repeated 100 times for each penalty value and the average MSE was computed. The optimal penalty was chosen as the one that generated the smallest average MSE. The model per imputed data set that corresponded to the optimal penalty was referred to as “best”. Also a “tolerance” model was considered by applying a stronger penalty that had an MSE within 3% of the optimum, yielding more parsimony. The final best and tolerance models comprised regression coefficients which were averaged over the ten imputed data sets. Therefore, if a covariate was chosen, for instance, in only one of the imputed data sets, its non-zero value was divided by 10, resulting in a smaller regression coefficient. Furthermore, since only the averaged model will be presented in practice, instead of 10 different models, we assessed the predictive value of the averaged best and tolerance models. This was done by checking their discriminative and calibrative performances.

#### Model performance: discrimination and calibration

**Figure 1:**
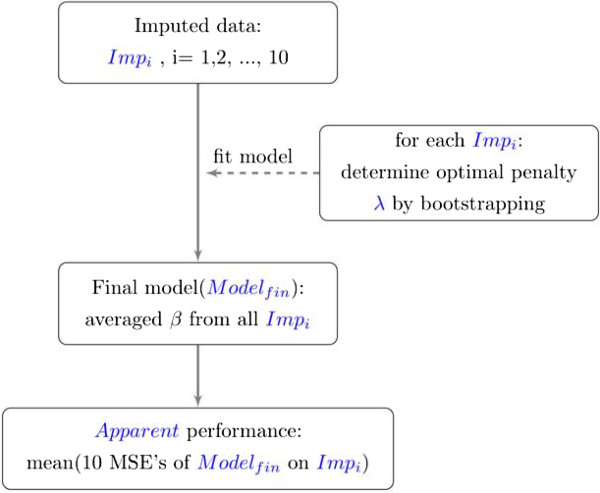
**A summary of the model construction steps and evaluation of performance via the lasso.**

The discriminative performance of the best and tolerance models was quantified with the MSE. Enumerated below (shown schematically in Figure 1) are steps to acquire an averaged apparent MSE over the multiply imputed data.

**Step 1**: Construct a model in all 10 imputed data sets (original samples: *Imp*_*i*_,*i* = 1,2,..,10) as described in the previous section, and average the regression coefficients over all data sets to obtain one final model, *Model*_*fin*._. **Step 2**: Using *Model*_*fin*._, determine the apparent performance on *Imp*_*i*_. This results in 10 apparent performances (*Apparent*_*i*_). **Step 3**: The final *Apparent* performance is the average over the 10 *Apparent*_*i*_ performances.

To assess calibration, the predicted CRQ dyspnea outcome scores were plotted against the observed values, along with their averages by deciles of predicted values. The corresponding calibration line was described using a linear regression with the observed outcome regressed on the linear predictor (LP): CRQ dyspnea = *α*_*LP*_+ *β*_*LP*_× LP. The parameter *β*_*LP*_ is termed calibration slope [24], which can be seen as a uniform shrinkage factor [10, 11]. A perfect calibration would yield a line with *α*_*LP*_= 0 and *β*_*LP*_= 1. For a clinically good calibration, the averages per decile should be within a +/-0.5 limit of the minimal clinically important difference [25].

#### Validation

Bootstrap resampling for internal validation and estimation of the expected optimism was performed based on Harrell [6]. This was performed to validate our averaged final model from the previous section. First, we considered validating the discrimination index, and below is a description of four approaches to handle the multiply imputed data sets in the validation procedure.

**Approach 1**: It is ensured that a bootstrap run selects the same subjects across the imputed data sets. Hence, bootstrap samples differ solely by the imputed values.

**Figure 2:**
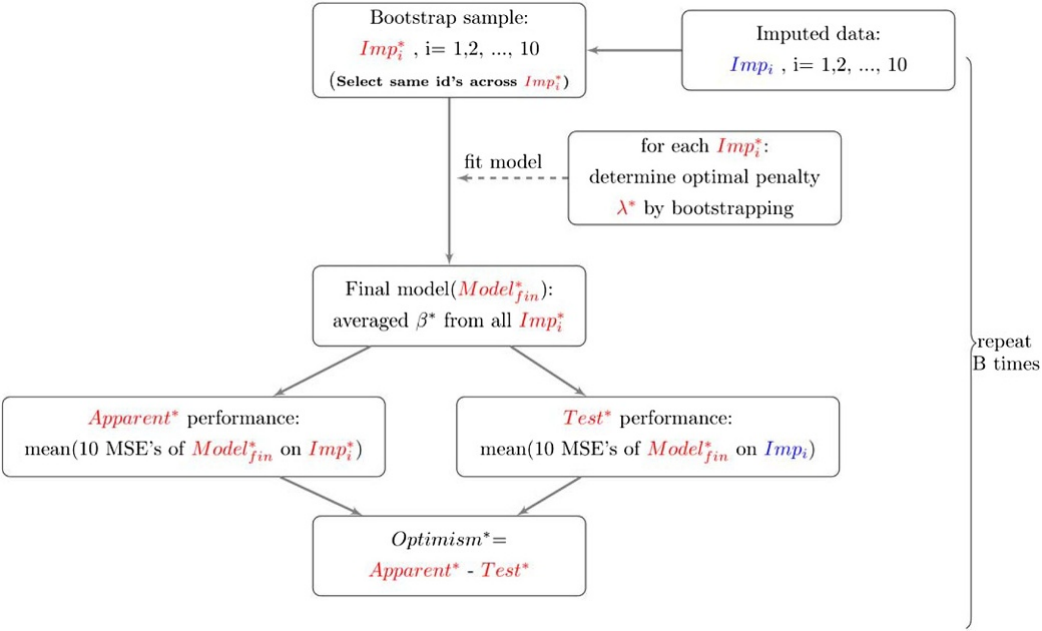
**An algorithm to calculate optimism in the presence of multiply imputed data based on Harrell** [6]**.** The *true performance* =*Apparent* (Step 3 in the Model performance: discrimination and calibration section) - mean (B *Optimism* ^∗^’s). B is the number of bootstrap samples and ^∗^ is used to denote bootstrap objects.

1. In a bootstrap draw select the same subjects over all *Imp*_*i*_ to get .
2. Redo every model building step from step 1 in the original model construction (previous section). The performance of  on each  is evaluated and averaged to obtain *Apparent*^∗^.
3. Apply  to the original samples, *Imp*_*i*_ to determine the averaged test performance, *Test*^∗^
4. Calculate the optimism, *Optimism*^∗^ as *Apparent*^∗^-*Test*^∗^
5. Repeat 1 to 4 at least 100 times to obtain a stable estimate of the optimism.
6. The optimism-corrected performance, *trueperformance*, is the difference of the *Apparent* (Step 3 in the Model performance: discrimination and calibration section) and mean of the 100 *Optimism*^∗^’s (estimated in 4).
  A schematic summary of approach 1 is shown in Figure 2. If there are no missing data, the same data are used in all 10 “subsamples”.

**Approach 2**: Records selected in a bootstrap run can differ over the imputed data sets. Thus as opposed to approach 1, subjects were not forced to be the same over the ’s.

**Approach 3**: Select just one of the imputed data sets and perform the resampling procedure as in the case where there is no missing data.

**Approach 4**: This approach differs from the previous approaches in that it incorporates the MI procedure in the validation. Here, for step 1, a bootstrap sample was taken from the incomplete data set and then MI was performed 10 times. The procedure then proceeded as described in step 2 to step 6 of approach 1.

The amount of miscalibration was quantified via the calibration slope *β*_*LP*_. Correction was achieved by re-estimating the intercept and multiplying each estimated effect with a shrinkage factor *s* [10, 11] that was determined as follows. In every bootstrap run, model construction per imputed data  was carried out as with the original sample *Imp*_*i*_ and values of the linear predictors LP ^∗^ were calculated on the original samples. The intercept ( and slope ( of LP ^∗^ was estimated by regressing the outcome in the original sample on the LP ^∗^. This process was repeated 100 times and *s* was calculated as the mean of the 100 estimates of  () [5, 6, 8]. The re-calibrated model was . Usually, , meaning that in the original model low predictions of the outcome will be too low and high predictions too high. In a case with over-shrinkage, , implying that low predictions of the outcome will be too high and high predictions too low. Multiplying each coefficient by  leads to shrinking (if ) or unshrinking (if ), which usually improves both calibration and MSE.

### Software

All analyses were implemented using the R statistical software, version 2.15.2 [26]. The mice package [21] was used to perform MI. Variable selection and model fitting was performed using the glmnet [27] and caret [28], packages. Additional routines were developed to perform the bootstrap resampling procedure in the presence of multiply imputed data (See Additional file 1).

## Results

**Figure 3:**
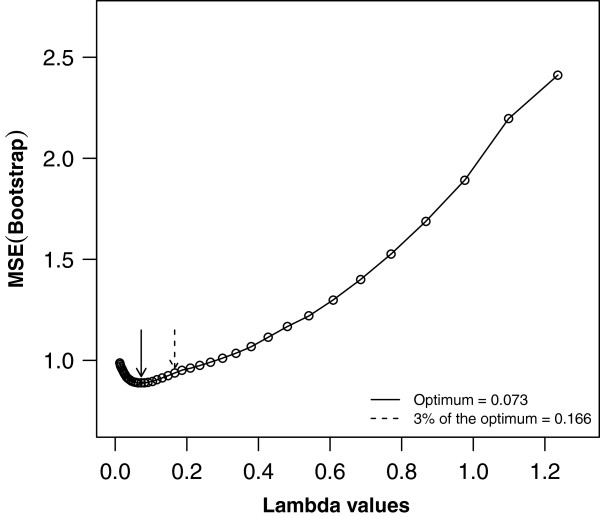
**Performance profile to determine the optimal lasso penalty tuning parameter (on one imputed data set) for a grid of 40 penalty values based on 100 bootstrap samples.** The optimal penalty value corresponding to the best model is that which generated the smallest average MSE over the bootstrap samples. A tolerance model can be estimated as that with MSE within 3% of the optimum in the direction of the stronger penalties.

In Figure 3, a summary of the parameter tuning procedure, showing the bootstrap performance of all 40 penalty values (on one imputed data set) is given. This illustrates that an optimal *λ* value was identifiable. The optimal lambda varied between 0.063 and 0.082 over the imputed data sets for the best model, and between 0.064 and 0.166 for the tolerance model. In Table 1 we report averaged coefficients of the best and tolerance models, and the number of times each variable was retained across the imputed data sets. In total, 19 and 10 covariates were retained at least once across the imputed data sets for the best and tolerance model respectively. The estimated optimism, calculated according to the four approaches described above, along with the apparent and optimism-corrected MSE’s are presented in Table 2. The estimate of optimism was sensitive to the choice of handling imputed data in the bootstrap procedure. Estimates from approach 1, 3 and 4 suggested that there was substantial optimism in the apparent performance. Larger values of optimism were observed with approach 4. On the other hand, approach 2 suggested there was very little or no optimism.

**Table 1:** **Models with the best penalty value and the penalty within 3% of the optimum**

Covariates		Best model parameters			3% tolerance model parameters	
	***Number***	***Uncalibrated β***	***Re-calibrated β***	***Number***	***Uncalibrated β***	***Re-calibrated β***
Intercept	10	1.038638	0.880085	10	1.30578	0.862149
Anxiety medication	10	-0.04399	-0.04534	1	-0.00066	-0.00075
Cardiovascular medication	10	-0.01752	-0.01774	0		
Pulmonary medication	10	-0.01454	-0.01486	0		
Use of long acting beta agonists	3	-0.00143	-0.00129	0		
Influenza vaccination	10	-0.04283	-0.04365	0		
Renal:chronic kidney disease	10	-0.06797	-0.06939	0		
Lung function	6	0.000162	0.00018	0		
HADS depression score	8	-0.00372	-0.00403	7	-0.00274	-0.00311
Physical activity score	10	0.02788	0.028764	6	0.005337	0.006068
Self-efficacy 2	3	0.01409	0.014715	3	0.006139	0.006978
Self-efficacy 3	1	0.000367	0.000384	0		
Sit to stand test	10	0.001774	0.001901	10	0.001518	0.001632
crqdyspnea	10	0.654104	0.682812	10	0.60923	0.690902
crqfatigue	10	0.109821	0.114304	10	0.089453	0.100929
crqmastery	6	0.010376	0.010272	1	0.001909	0.002171
crqemotional	3	0.00078	0.00089	2	0.00134	0.001523
Feeling thermometer change score	10	-0.04587	-0.04658	0		
HADS depression change score	10	0.039783	0.040061	0		
crqdyspnea change score	10	-0.1582	-0.16403	10	-0.04866	-0.05207

The column “number” gives the number of times each variable was selected across the imputed data sets.

**Table 2:** **Apparent and optimism-corrected MSE values based on the four approaches (“Appr 1”, “Appr 2”, “Appr 3” and “Appr 4”) of handling multiply imputed data sets, and the shrinkage factor (**  **)**

MSE _0_= 2.4183		Averaged best model 0.9047				Averaged 3% tolerance 0.9672		
Apparent MSE _***X***_	***Appr 1***	***Appr 2***	***Appr 3***	***Appr 4***	***Appr 1***	***Appr 2***	***Appr 3***	***App 4***
Optimism	-0.1162	-0.0127	-0.0988	-0.1452	-0.0656	-0.0081	-0.0537	-0.0781
Optimism corrected MSE _*X*_	1.0209	0.9174	1.0035	1.0499	1.0328	0.9753	1.0209	1.0453
	1.0443	1.0552	1.0361	1.0498	1.1368	1.1257	1.1136	1.1297

**Figure 4:**
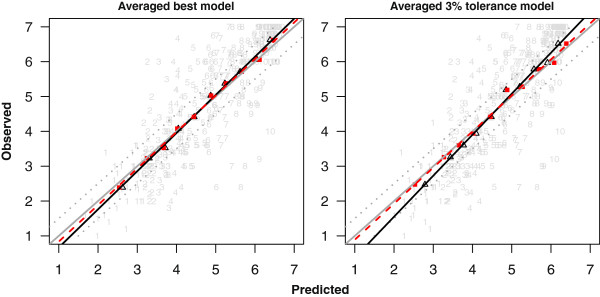
**Plots of the observed against the predicted CRQ dyspnea (range from 1 (worst) to 7 (best)) at 12 months.** The gray diagonal line represents perfect calibration. The black solid line and red dashed lines are the regression lines for uncalibrated and re-calibrated models. Black open triangular points and red filled square points are based on deciles of predicted CRQ dyspnea from the uncalibrated and re-calibrated models respectively. The dotted gray lines represent the +/-0.5 minimal clinically important difference. The raw data is represented by the gray points. The numbers indicate deciles to which they belong.

The calibration plots in Figure 4 show that there was over-shrinkage of the coefficients. This was more obvious with the tolerance model, which is to be expected since a stronger penalty was applied. The *β*_*LP*_’s were greater than 1 (as shown by the solid black lines in Figure 4). Similar estimates of  were obtained over the four approaches (2). The calibration was improved after re-calibrating the coefficients. This is shown (for the case where  was estimated via approach 1) by the dashed red lines in Figure 4. The re-calibrated coefficients are also provided in Table 1.

## Simulation study

### Study setup

We simulated 20 covariates *X*_*j*_ from a multivariate normal distribution with *μ*_*j*_= 0 and *σ*_*j*_= 1, for j = 1,2…, 20. Most of the correlations were zero except for *R*_1,5_ = 0.72,*R*_1,6_ = - 0.52,*R*_2,8_ = 0.74,*R*_4,12_ = - 0.82,*R*_6,16_ = - 0.34,*R*_10,20_ = - 0.38,*R*_11,19_ = 0.37,*R*_19,20_ = 0.65. *X*_1_ to *X*_10_ were categorized as binary covariates. Dichotomization of *X*_1_,*X*_2_,*X*_6_ and *X*_7_ was at their respective 50th percentile values. The categories for *X*_3_,*X*_4_,*X*_8_ and *X*_9_, and for *X*_5_ and *X*_10_, was at their 30th and 20th percentile values respectively. The regression coefficients were taken to be *β*_0_ (intercept) = 1.14,*β*_1_ to *β*_5_ = 0, *β*_6_ = - 0.839,*β*_7_ = 1.131,*β*_8_ = - 1.540,*β*_9_ = 1.426,*β*_10_ = 0.854,*β*_11_ to *β*_15_ = 0,*β*_16_ = 0.457,*β*_17_ = - 0.494,*β*_18_ = - 0.738,*β*_19_ = 1.589,*β*_20_ = 0.845. The outcome was *Y* = *β*_0_ + *X*^*T*^*β* + *ε*, where *ε* ∼ *N* (0,*sd* = 1.74). Nine out of the 20 covariates always contain missing values which were missing completely at random, with percentage of missing values based on draws from a binomial distribution; *X*_2_,*X*_7_,*X*_12_ and *X*_17_ with a 20% chance of missing, and *X*_3_,*X*_8_,*X*_13_ and *X*_18_ with a 50% chance of missing. There were no missing outcome data. For every simulated data set the following procedures were performed.

1. A lasso linear regression model with all covariates was fitted to the data in the setting without missing values (NM).
2. In the setting with missing data (WM), missing values were imputed 10 times using MICE and a lasso linear regression model was fitted to each imputed data set. Similar to the study data, the final model was an average of coefficients over the imputed data sets. Hence covariates that were estimated to be zero several times had small coefficients.
3. The expected optimism, referred to as *Optimism*_*internal*_, was estimated in the NM and WM settings respectively via bootstrap resampling as described by Harrell [6] and the four approaches described above. For both settings, the final models were evaluated on the original sample and on a new independent data set (with no missing values) to obtain *MSE*_*apparent*_ and *MSE*_*external*_ respectively. The observed optimism (*Optimism*_*external*_) was the difference between *MSE*_*apparent*_ and *MSE*_*external*_. We would expect *Optimism*_*internal*_ and *Optimism*_*external*_ to be close if the resampling procedure gives unbiased estimates of optimism.

### Simulation study results

**Figure 5:**
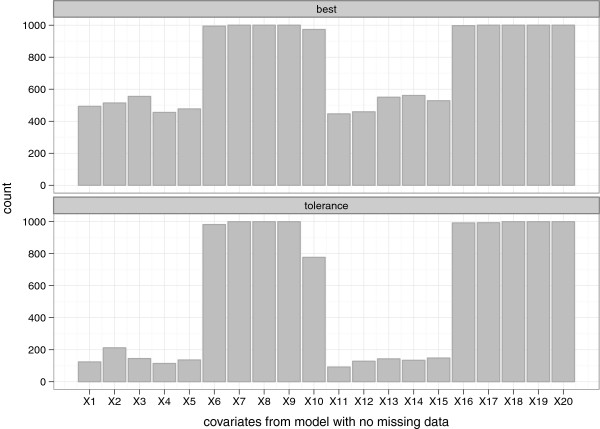
**Frequency of selection per covariate from the data without missing values (NM).** “best” and “tolerance” correspond respectively to models with the optimal penalty (smallest MSE) and a penalty that has MSE within 3% of the optimum.

**Figure 6:**
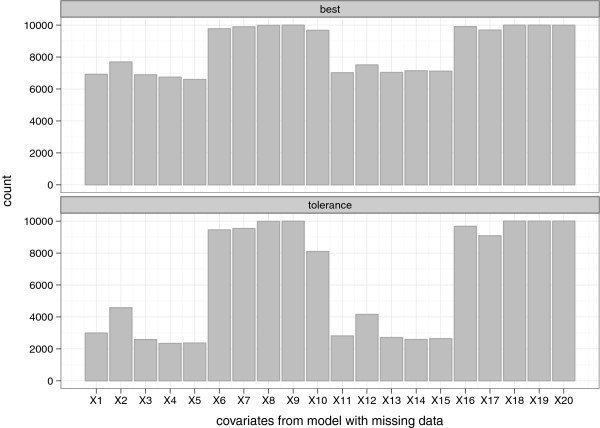
**Frequency of selection per covariate (retained at least once across the 10 imputed data sets) from the data with missing values (WM).** “best” and “tolerance” correspond respectively to models with the optimal penalty (smallest MSE) and a penalty that has MSE within 3% of the optimum.

**Figure 7:**
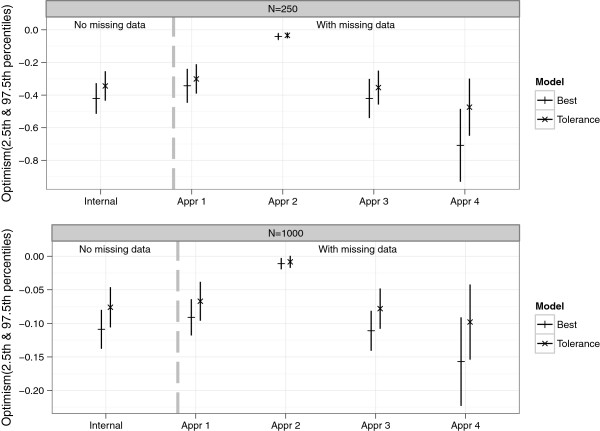
**Estimates of optimism from the simulation study.** Optimism “Internal” was the difference between the bootstrap performance (on bootstrap data) and the test performance (on original data). Optimism “Appr 1”, “Appr 2”, “Appr 3” and “Appr 4” were based on the four approaches of handling missing data.

**Figure 8:**
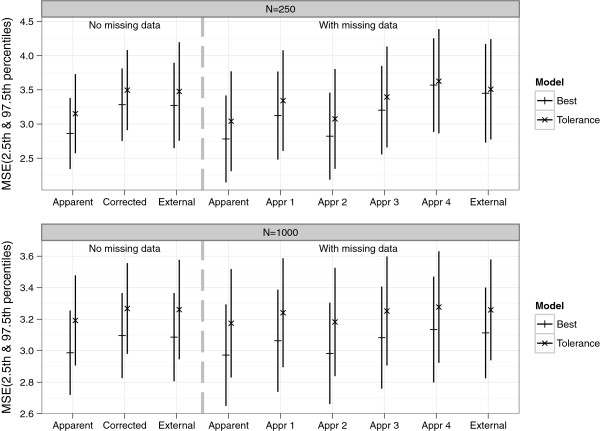
**Means of estimated MSE values from the simulation study.** MSE “Apparent” was the performance on the original data. MSE “Corrected” = *MSE*_*apparent*_- *Optimism*_*internal*_ MSE “External” was the performance on an independent new data with no missing values MSE “Appr 1”, “Appr 2”, “Appr 3” and “Appr 4” were based on subtracting the value of optimism estimated via the four approaches of handling missing data from *MSE*_*apparent*_ (with missing data).

**Figure 9:**
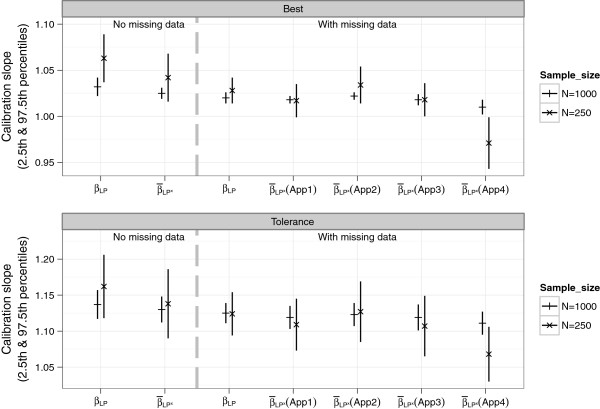
**Means of estimated of calibration slope parameters from the simulation study.** *β*_*LP*_ was the slope of the the linear predictor (LP) estimated from regressing the observed outcome on LP from the original data.  (shrinkage factor) was the slope of *L* *P* ^∗^ estimated by regressing the outcome in the original sample on the *L* *P* ^∗^ from the bootstrap sample.

We used sample sizes of *n* = 250 and 1000, and performed 1000 simulations. The results are summarized in Figures 5, 6, 7, 8 and 9, and in Additional file 2: Table S1 which shows the means and corresponding 2.5th and 97.5th percentile values within parentheses.

Figures 5 and 6 give an impression of how frequent the relevant (*β*_*j*_≠ 0) and the irrelevant (*β*_*j*_= 0) covariates were selected across the simulated data sets, with *n*=250. As earlier observed [29], the best model retained a large number of irrelevant covariates (*X*_1_ to *X*_5_ and *X*_11_ to *X*_15_), with the selection frequency ranging from about 45% to 55% for the NM setting. This was higher for the WM setting (66% to 75%) because covariates were counted if they were included in at least one of the imputed date sets. A more desirable selection was achieved with the tolerance models; selection frequency of the irrelevant covariates in the range 11% to 20% and 25% to 47% for the NM and WM settings respectively. Similar findings were observed with *n* = 1000, where much lower selection frequencies of the irrelevant covariates were observed for the tolerance model; ranging from 0% to 8 *%* and 3% to 25% for the NM and WM settings respectively. Notice that the relevant covariates were selected much more frequently (selection frequency generally ranging from about 79% to 100%) than the irrelevant ones. In the NM setting, the lasso was able to find the correct model (that is with respect to selecting all the relevant predictors simultaneously) 97.1% and 76.8% of the time for the best and tolerance models respectively. It was only one relevant variable off in about 2.9% and 22.2% of the time for the best and tolerance models respectively. In the WM setting, considering the case where variables were retained in at least 50% of the imputed data sets, the correct model was selected about 97.6% and 75.7% of the time for the best and tolerance models respectively. It was only one relevant variable off in about 2.3% and 21.5% of the time for the best and tolerance models respectively.

For both the NM and WM settings, the apparent MSE’s were optimistic. Estimates of optimism along with 2.5th and 97.5th percentiles are shown in Figure 7 for both NM and WM settings. In the NM setting and with *n* = 250, the mean *Optimism*_*internal*_-0.421 (best model) and -0.344 (tolerance model) were close to but significantly different from the mean *Optimism*_*external*_-0.410 and -0.324 for best and tolerance models respectively. With a larger sample size, *n* = 1000, the mean *Optimism*_*internal*_ and *Optimism*_*external*_ were more similar and had smaller 2.5th and 97.5th percentile values (see Additional file 2: Table S1). In the WM setting, the mean *Optimism*_*internal*_ differed significantly between the four approaches of handling imputed data in the validation procedure for both *n* = 250 and 1000. Histograms of *Optimism*_*internal*_ from these approaches are shown in Additional file 3: Figure S1 (*n* = 250). While the estimates from applying approach 1, 3 and 4 suggested that there was optimism in the apparent performance, findings from approach 2 on the other hand suggested little or no optimism. Similar to the real data example, larger values of optimism were obtained via approach 4. The mean *Optimism*_*internal*_ based on approach 3 (best = - 0.421, tolerance = - 0.354) was very similar to that obtained from the NM setting (best = - 0.421, tolerance = - 0.344), because both used just a single data set. Also, the mean *Optimism*_*internal*_ from approach 1 (best = - 0.343, tolerance = - 0.301) and approach 3 were smaller than the mean *Optimism*_*external*_ (best = - 0.668, tolerance = - 0.467), while that from approach 4 (best = - 0.708, tolerance = - 0.474) was slightly larger. However with a larger sample *n* = 1000, apart from approach 2 which still suggested negligible optimism, the mean *Optimism*_*internal*_ and *Optimism*_*external*_ were much more similar for both the NM and WM settings (see Additional file 2: Table S1). Figure 8 summarizes the estimated MSE values. Compared to the NM setting, the *MSE*_*external*_ was larger for the WM setting (probably because more irrelevant covariates were retained).

There was suboptimal calibration due to over-shrinkage, apparent from the mean *β*_*LP*_’s which were > 1 in both the NM and WM settings with both *n*=250 and 1000. As expected there was more shrinkage with the tolerance (tol) model since it applied a stronger penalty. The means of  also differed between the four approaches of handling imputed data in the resampling procedure. Estimates from all 4 approaches were more similar to each other for the larger sample size *n* = 1000 (see Figure 9 and Additional file 2: Table S1).

## Discussion

We constructed models to predict CRQ dyspnea 6 months ahead for a cohort of COPD patients by using the lasso technique. This approach combines shrinkage and variable selection, and is promising when prediction and parsimony are goals of predictive modelling. It can also be applied to generalized linear models such as the logistic or Cox model [1, 2]. Multiple imputation (MI) was implemented to cater for incomplete data, and the optimal lasso penalty for each imputed data set was determined via bootstrapping.

Nineteen predictors were retained by the best model (with the optimal penalty), which may be an unreasonable large number of predictors to use in practice. A stronger penalty can be applied to pick a smaller subset of covariates without sacrificing too much performance. Here for instance we chose a stronger penalty that had the MSE within 3% of the optimum, resulting to a reduced subset of 10 covariates. Another approach would be to apply a “majority method” that selects only variables that were retained in all imputed data sets. However, though parsimony is desired for practice, it is often at the expense of a lower predictive performance. On the other hand the lasso prevents overfitting. But the large variability in the set of selected predictors as demonstrated in our simulation study and earlier by Van Houwelingen [29] is unappealing. It was observed in our simulation study that the final best models often retained all the relevant covariates but were also commonly contaminated with irrelevant covariates, especially in the WM settings. There was less contamination with the tolerance models.

The calibration plots showed that there was over-shrinkage of coefficients. Also, applying the lasso resulted in optimistic estimates of model performance. This implies that the lasso penalty chosen via bootstrapping was optimal only for the data at hand. The same was observed in the simulation study for both the setting with (NM) and without (WM) missing data. Thus, as most model building procedures, the model constructed via the lasso still requires validation. Moreover, it has the tendency of retaining redundant covariates. This was more frequent for WM settings since different variables could be selected for different imputed data sets. Some ways to handle variable selection over multiply imputed data sets have been addressed in the literature [30–33]. The multiple imputation lasso (MI-LASSO), which applies a group lasso penalty, has been proposed to select the same variables across multiply-imputed data sets [31]. A comparable level of parsimony and model performance was observed between the MI-LASSO model and our tolerance model with both the real data and the simulated data sets. In the simulation study, we observed that the frequency of selection of the relevant (*X*_6_ to *X*_10_ and *X*_16_ to *X*_20_) and irrelevant (*X*_1_ to *X*_5_ and *X*_11_ to *X*_15_) covariates using the MI-LASSO technique was very similar to that obtained using our tolerance model. The selection frequency of the irrelevant covariates was in the range of 25% to 47% for our tolerance model and 25% to 65% for the MI-LASSO. The mean *MSE*_*corrected*_ (*MSE*_*external*_ within parentheses) over the 1000 simulated data set (n = 250, approach = 4) was 3.559 (3.440) and 3.626 (3.508) for the MI-LASSO and our tolerance model respectively.

The estimate of optimism was sensitive to the choice of handling the imputed data sets in the bootstrap resampling procedure. This was observed with both the study and simulated data sets. The results based on approach 1, 3 and 4 suggested that the lasso models were optimistic.

Approach 1, 2 and 3 were performed by resampling data sets that had been completed via MI, and an important question was how to sample subjects over the imputed data sets. Approach 1 ensured that for a bootstrap draw, the samples from each imputed data set differed only by the imputed values as in the original data sets. In approach 2, bootstrap samples over the imputed data could differ by the imputed values as well as the selected subjects. This led to an underestimation of optimism (discussed in the next paragraph). Approach 3 performed the validation procedure using only one imputed data set. This was easier to perform as it mimicked the procedure where there was no missing data, and required less bookkeeping. Approach 4 on the other hand resampled the incomplete data set and then applied MI, thus incorporating the MI procedure in the validation. In the simulation study, the mean *Optimism*_*internal*_ from approach 1 was significantly smaller than that from approach 3, and both were biased downward with respect to the mean *Optimism*_*external*_. In the case of approach 4, the mean *Optimism*_*internal*_ was more similar to *Optimism*_*external*_, though slightly biased upward. The same was observed with the NM setting. A more upward bias was observed when approach 4 was performed such that only one imputation was derived from a bootstrap sample taken from the original data with missing values. So we advise to use multiple imputations. The biases from approach 1, 3 and 4 were smaller for *n* = 1000, where a smaller number of irrelevant covariates were retained compared to when n = 250, and were much smaller with the tolerance models.

Contrary to approach 1, 3 and 4, approach 2 clearly underestimated optimism since *MSE*_*corrected*_ for both *n* = 250 and 1000 were lower than *MSE*_*external*_, and even lower than the theoretical MSE (1.74^2^= 3.028). The explanation is that a bootstrap draw across the imputed data sets differed by both the imputed values as well as the selected subjects, introducing more heterogeneity between the bootstrap imputed data sets than there should be. Consequently a more robust final model (that averaged coefficients from all 10 data sets) was achieved. The performance of this model was similar in both the bootstrap and the original samples, leading to a negligible estimate of optimism as observed with both the real data and in the simulation study. Approach 2 was repeated using 10 copies of the same data with no missing values, mimicking a set-up with 10 multiply imputed data. This still gave very small values of *Optimism*_*internal*_, which wrongly suggested negligible optimism.

## Conclusions

We advise that prognostic models constructed via the lasso technique should also be evaluated for optimism. When data are missing and resampling techniques are used to estimate optimism, it does matter how multiply imputed data sets are handled. Improper handling of the multiply imputed data sets might results in substantially large underestimation of optimism as is the case with approach 2. We recommend approach 4 since it fully replays every step that was performed with the original data with missing values, and yields estimates of optimism that are close to the *Optimism*_*external*_.

## Electronic supplementary material

Additional file 1: **R function to perform resampling with caret package in the presence of multiply imputed data.** The “validate.train” function below estimates optimism in predictive value via the bootstrap resampling procedures described in approach 1 and 4 in the manuscript. In approach 1 the completed data sets (via MI) are resampled. The same subjects are selected across the imputed data sets so that the bootstrap imputed data sets always differ only by their imputed values. In approach 4, the incomplete data set is resampled and then MI is performed using the mice package. The function can be used to estimate optimism in the predictive value of a linear regression model constructed within caret using the train() function, with method = “glmnet”. In order to be consistent with the output from caret, we assumed that the response variable is always in the last column of every data set. (ZIP 2 KB)

Additional file 2: Table S1: Simulation study results. The table presents means of all estimates along with their corresponding 2.5th and 97.5th percentile values within parentheses. These are based on 1000 simulated data sets for both n = 250 and 1000. (PDF 32 KB)

Additional file 3: Figure S1: Distribution of the estimated expected optimism values from the simulation study. These are based on 1000 simulated data sets (n = 250) for both the setting without (NM) and with (WM) missing data. (ZIP 6 KB)
