# Supplementary material for: Validation of prediction models based on lasso regression with multiply imputed data
Source: BMC Med Res Methodol. 2014 Oct 16;14:116. doi: 10.1186/1471-2288-14-116 (PMC4209042; doi:10.1186/1471-2288-14-116)
Supplement: Supplementary file 2 — Additional file 2: Table S1: Simulation study results. The table presents means of all estimates along with their corresponding 2.5th and 97.5th percentile values within parentheses. These are based on 1000 simulated data sets for both n = 250 and 1000. (PDF 32 KB) [file 12874_2014_1128_MOESM2_ESM.pdf]

Table Additional file 2: Table S1. Simulation study results based on 1000 data sets (n = 250 and 1000). Means of all estimates along with their corresponding 2.5th and 97.5th percentile values within parentheses. The theoretical MSE is 1.74^2= 3.028.

Estimates	Without missing data		With missing data							
	**n = 250**									
	best	*tol*					*best*	*tol*		
$MSE_{apparent}$	2.862(2.347;3.427)	3.152(2.588;3.799)					2.782(2.121;3.411)	3.041(2.289;3.770)		
$MSE_{external}$	3.272(2.687;3.929)	3.476(2.858;4.234)					3.450(2.787;4.210)	3.508(2.866;4.315)		
$Optimism_{external}$	-0.411(-1.199;0.357)	-0.324(-1.205;0.538)					-0.668(-1.699;0.182)	-0.467(-1.537;0.427)		
β_{LP}	1.063(1.042;1.088)	1.162(1.123;1.206)					1.042(1.018;1.067)	1.138(1.092;1.186)		
	best	*tol*	*Appr 1 best*	*Appr 1 tol*	*Appr 2 best*	*Appr 2 tol*	*Appro 3 best*	*Appr 3 tol*	*Appr 4 best*	*Appr 4 tol*
$Optimism_{internal}$	-0.421(-0.521;-0.334)	-0.344(-0.442;-0.264)	-0.343(-0.444 -0.243)	-0.301(-0.393;-0.220)	-0.041(-0.060;-0.024)	-0.034(-0.054;-0.017)	-0.421(-0.540;-0.307)	-0.354(-0.461;-0.258)	-0.708(-0.826;-0.566)	-0.474(-0.510;-0.389)
$MSE_{corrected}$	3.283(2.746;3.868)	3.496(2.927;4.143)	3.124(2.422;3.795)	3.342(2.551;4.084)	2.822(2.159;3.472)	3.075(2.309;3.813)	3.203(2.486;3.864)	3.395(2.610;4.157)	3.570(3.173;3.993)	3.626(3.098;4.188)
$\bar{\beta}_{LP^*}$	1.028(1.013;1.041)	1.124(1.097;1.155)	1.017(1.001;1.033)	1.109(1.072;1.145)	1.034(1.016;1.052)	1.127(1.087;1.170)	1.018(1.003;1.038)	1.107(1.067;1.151)	0.971(0.942;0.996)	1.068(1.031;1.108)
	**n = 1000**									
	best	*tol*					*best*	*tol*		
$MSE_{apparent}$	2.987(2.733;3.253)	3.192(2.921;3.477)					2.972(2.665;3.288)	3.174(2.838;3.515)		
$MSE_{external}$	3.086(2.823;3.360)	3.261(2.960;3.561)					3.113(2.845;3.392)	3.259(2.964;3.576)		
$Optimism_{external}$	-0.099(-0.460;0.281)	-0.069(-0.430;.321)					-0.141(-0.543;0.278)	-0.085(-0.482;0.350)		
β_{LP}	1.032(1.021;1.041)	1.137(1.118;1.156)					1.025(1.020;1.032)	1.130(1.112;1.147)		
	best	*tol*	*Appr 1 best*	*Appr 1 tol*	*Appr 2 best*	*Appr2 tol*	*Appr 3 best*	*Appr 3 tol*	*Appr 4 best*	*Appr 4 tol*
$Optimism_{internal}$	-0.109(-0.138;-0.082)	-0.076(-0.105;-0.048)	-0.091(-0.119;-0.065)	-0.067(-0.093;-0.039)	-0.011(-0.019;-0.003)	-0.008(-0.017;0.001)	-0.111(-0.141;-0.081)	-0.078(-0.107;-0.048)	-0.157(-0.234;-0.103)	-0.098(-0.164;-0.053)
$MSE_{corrected}$	3.096(2.828;3.381)	3.268(2.985;3.564)	3.063(2.748;3.395)	3.241(2.903;3.597)	2.983(2.676;3.297)	3.182(2.846;3.523)	3.083(2.761;3.422)	3.252(2.910;3.603)	3.144(2.967;3.356)	3.290(3.085:3.530)
$\bar{\beta}_{LP^*}$	1.020(1.014;1.026)	1.125(1.111;1.140)	1.018(1.013;1.022)	1.119(1.103;1.135)	1.022(1.019;1.025)	1.123(1.106;1.140)	1.018(1.013;1.024)	1.119(1.100;1.137)	1.010(1.002;1.018)	1.111(1.096;1.126)

"Appr 1", "Appr 2", "Appr 3"and "Appr 4" are respectively the four approaches to handle missing data.

"best" and "tol" stand for the model with optimal penalty value and that within 3% of the optimal model respectively

$MSE_{apparent}$ was the performance on the original data

$MSE_{external}$ was the performance on an independent new data with no missing values

$Optimism_{internal}$ was the difference between the bootstrap performance (on bootstrap data) and the test performance (on original data)

$Optimism_{external} = MSE_{apparent} - MSE_{external}$

$MSE_{corrected} = MSE_{apparent} - Optimism_{internal}$

β_{LP} was the slope of the the linear predictor (LP) estimated from regressing the observed outcome on LP from the original data

$\bar{\beta}_{LP^*}$ was the slope of LP^* estimated by regressing the outcome in the original sample on the LP^* from the bootstrap sample.
